# An Integrative Nomogram for Identifying Early-Stage Parkinson's Disease Using Non-motor Symptoms and White Matter-Based Radiomics Biomarkers From Whole-Brain MRI

**DOI:** 10.3389/fnagi.2020.548616

**Published:** 2020-12-17

**Authors:** Zhenyu Shu, Peipei Pang, Xiao Wu, Sijia Cui, Yuyun Xu, Minming Zhang

**Affiliations:** ^1^Department of Radiology, Zhejiang Provincial People's Hospital, Affiliated People's Hospital of Hangzhou Medical College, Hangzhou, China; ^2^GE Healthcare China, Shanghai, China; ^3^Department of Radiology, The Second Affiliated Hospital, Zhejiang University School of Medicine, Hangzhou, China; ^4^Second Clinical College, Zhejiang Chinese Medical University, Hangzhou, China

**Keywords:** radiomics, white matter, magnetic resonance imaging, machine learning, Parkinson's disease

## Abstract

**Purpose:** To develop and validate an integrative nomogram based on white matter (WM) radiomics biomarkers and nonmotor symptoms for the identification of early-stage Parkinson's disease (PD).

**Methods:** The brain magnetic resonance imaging (MRI) and clinical characteristics of 336 subjects, including 168 patients with PD, were collected from the Parkinson's Progress Markers Initiative (PPMI) database. All subjects were randomly divided into training and test sets. According to the baseline MRI scans of patients in the training set, the WM was segmented to extract the radiomic features of each patient and develop radiomics biomarkers, which were then combined with nonmotor symptoms to build an integrative nomogram using machine learning. Finally, the diagnostic accuracy and reliability of the nomogram were evaluated using a receiver operating characteristic curve and test data, respectively. In addition, we investigated 58 patients with atypical PD who had imaging scans without evidence of dopaminergic deficit (SWEDD) to verify whether the nomogram was able to distinguish patients with typical PD from patients with SWEDD. A decision curve analysis was also performed to validate the clinical practicality of the nomogram.

**Results:** The area under the curve values of the integrative nomogram for the training, testing and verification sets were 0.937, 0.922, and 0.836, respectively; the specificity values were 83.8, 88.2, and 91.38%, respectively; and the sensitivity values were 84.6, 82.4, and 70.69%, respectively. A significant difference in the number of patients with PD was observed between the high-risk group and the low-risk group based on the nomogram (*P* < 0.05).

**Conclusion:** This integrative nomogram is a new potential method to identify patients with early-stage PD.

## Introduction

Parkinson's disease (PD) is a common age-related progressive neurodegenerative disease (Dorsey et al., 2007). PD begins subtly and progresses slowly; thus, when the diagnosis is clear, most patients are in the middle or late stages of the disease. As the use of neuroprotective drugs by these patients has little effect on the speed of PD progression (LeWitt, 2015), an early diagnosis is of paramount importance for identifying disease onset and developing effective treatment plans. Currently, the diagnosis of PD mainly depends on the patient's medical history and clinical symptoms; however, the early stages of PD can include many atypical symptoms such as sleep disorders, decreased olfactory function and cognitive disturbances, and these non-motor symptoms often precede clinical motor signs (Mielke and Maetzler, 2014). Unfortunately, the cardinal and defining nonmotor symptoms used for the early diagnosis of PD in the clinic, particularly the symptoms that are typical of the early stages, also occur in patients with other disorders (Trojano and Papagno, 2018; De Pablo-Fernández et al., 2019), and the diagnostic error rate is as high as 25% among practitioners with limited clinical experience in diagnosing early-stage of PD (Miller and O'Callaghan, 2015). Thus, it is very challenging to diagnose early-stage PD based on current diagnostic standards.

In recent years, progress has been achieved in magnetic resonance imaging (MRI) technology in the field of neuroimaging (Agosta et al., 2017), such as structural MRI (Tzarouchi et al., 2010), diffusion tensor imaging (Schwarz et al., 2013), and blood oxygen level-dependent functional MRI (Benzagmout et al., 2019). These different techniques represent effective methods for non-invasively studying changes in brain morphology and function associated with PD. However, given the high cost of long functional imaging sessions and a general lack of standard imaging protocols, as represented by differences between MRI systems, scanning protocols and magnetic field strength (Frederick and Meijer, 2014), these complex scanning techniques cannot become widespread in clinical practice. Therefore, the identification of a simple, noninvasive measure to preclinically identify patients with early-stage PD is important.

Radiomics is a recently emerged field of radiology that quantifies imaging data with the aid of advanced image processing techniques, including high-throughput analysis and feature selection, to build biomarkers for the complete characterization of tumors (Liu et al., 2019). At this stage, the new quantitative imaging technology of radiomics has already been used to diagnose neurodegenerative diseases, including PD (Shinde et al., 2019). Nevertheless, most PD studies using radiomics examine only the substantia nigra (SN), where iron accumulation is spatially heterogeneous, allowing clinicians to easily distinguish patients with PD from healthy people (Guan et al., 2017). While the SN provides robust disease biomarkers, the concern is that radiomic analysis of the SN depends on the use of a special sequence, such as quantitative susceptibility mapping or neuromelanin-sensitive imaging, to display the contours of the SN. Understandably, the complexity of this technology has limited its clinical application.

However, the SN of patients with PD is not the only area exhibiting obvious disease-related tissue changes. White matter (WM) has also exhibits been reported to exhibit widespread microstructural alterations in patients with early-stage PD in the absence of gray matter atrophy and cognitive impairment (Pelizzari et al., 2020). In addition, several studies have shown widespread WM and gray matter changes in individuals with PD (Muthuraman et al., 2017; Koirala et al., 2019). Hence, an investigation of WM microstructural integrity in patients with PD may enable more successful exploration of early biomarkers of PD. Previous studies based on diffusion tensor imaging have shown that patients with PD present a greater decrease in WM integrity (Pozorski et al., 2018) than healthy people and often display extensive changes in the microstructure of WM in the early stage of PD even before the onset of cortical neuron loss (Rektor et al., 2018). Based on this evidence, structural changes occur earlier than physiological changes in the early stage of PD. Moreover, a three-dimensional radiomic analysis of WM throughout the brain was recently shown to reflect microstructural changes based on conventional T1-weighted imaging sequences (Shu et al., 2020), which may be more suitable than diffusion tensor imaging sequences for clinical application based on cost alone. Accordingly, we hypothesized that the structural changes in WM in patients with early-stage PD would also be detected by a radiomic analysis and would be of potential use for exploring new imaging-based disease biomarkers. To the best of our knowledge, this type of analysis has not yet been performed.

PD is a complex neurodegenerative disorder in which many different pathophysiological processes have been identified in different brain regions. Furthermore, a single WM biomarker will not be able to accurately diagnose and monitor disease progression; rather, a combination of different biomarkers should be used to provide a more comprehensive approach. As shown in previous studies, early diagnosis has been accomplished by the detection of multiple factors including impaired olfaction, depression, rapid eye movement sleep behavior disorder (RBD), excessive daytime sleepiness (EDS) and cognitive decline (CD) (Kalia and Lang, 2015), which usually occur in the prodromal stage of PD (Filippi et al., 2018). Accordingly, the purpose of this study was to explore the possibility of developing novel imaging biomarkers of PD from WM using radiomics and combining them with prodromal nonmotor symptoms to generate an integrative nomogram for disease classification. Overall, we hope to propose a low-cost and highly accurate method for identifying patients with early-stage PD.

## Materials and Methods

### Patients

**Figure 1:**
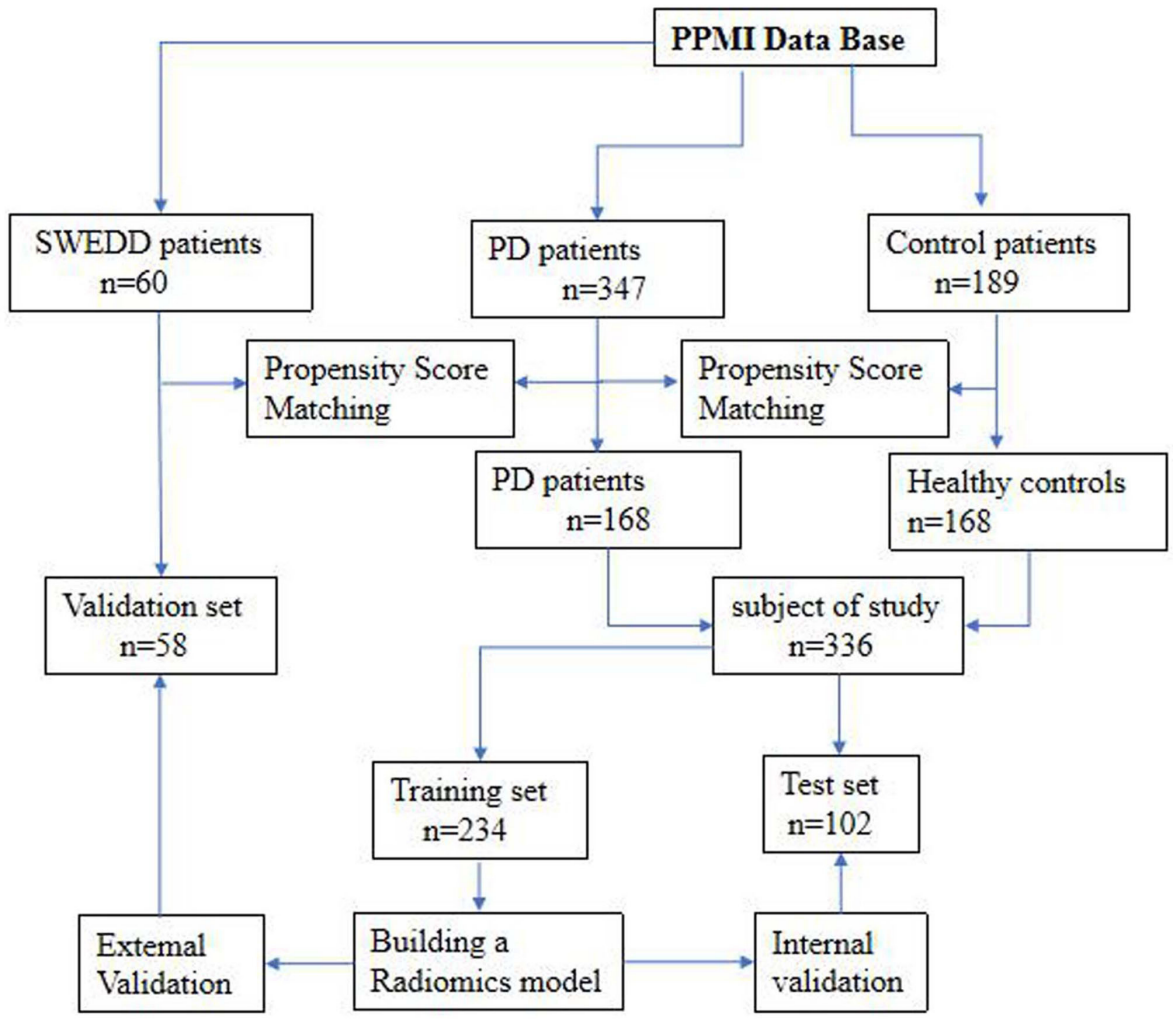
Flowchart of the recruitment process in the current study.

The datasets used to build the model were all obtained from the Parkinson's Progress Markers Initiative (PPMI) database (http://www.ppmi-info.org), which is the first global and comprehensive international Parkinson's research database (Parkinson Progression Marker Initiative, 2011). The PPMI is a landmark observational clinical study designed to comprehensively evaluate cohorts of significant interest using advanced imaging, biological, clinical and behavioral assessments to identify biomarkers of PD progression. Because the PPMI is a longitudinal study, we chose to use the baseline data to study early-stage PD. Importantly, all subjects were in the first or second stage of the disease according to the Hoehn-Yahr scale, and none of them had received drug treatment. The average interval between the development of clinical symptoms and the diagnosis of PD in these patients was 16.5 ± 14.9 months. Therefore, we defined the disease stage of these subjects as the early stage of PD. The detailed characteristics of the patients and the disease duration are provided in the Supplementary Materials. After age and sex matching, 168 healthy controls (HCs) and 168 patients with PD were selected from the database. These 336 subjects were then randomly divided into a training set (*n* = 234) and a test set (*n* = 102). The training set was used to build the diagnostic model, and the test set was used to verify the reliability of the model. We also investigated 58 age- and sex-matched patients with atypical PD from the PPMI who had imaging scans without evidence of dopaminergic deficit (SWEDD) to determine whether the model was able to distinguish patients with typical PD from patients with SWEDD. The matching details are provided in the supporting materials. Figure 1 shows the recruitment process for the research study.

### Whole-Brain White Matter Segmentation and Image Preprocessing

We obtained T1-weighted MRI data from the PPMI database. The PPMI diffusion MRI data were acquired using Siemens Tim Trio and Siemens Verio 3 Tesla MRI scanners at 32 different international sites based on a standardized protocol. T1-weighted MRI data were obtained using the following parameters: TR = 2300 ms, TE = 2.98 ms, TI = 900 ms, image matrix = 240 × 256 × 176, and voxel resolution = 1 × 1 × 1 mm^3^. All images were automatically segmented into whole-brain gray matter, WM and cerebrospinal fluid volumes using the spm12 software package (https://www.fil.ion.ucl.ac.uk/spm/software/spm12/). The WM boundaries were manually adjusted using ITK-SNAP software (http://www.itksnap.org) by two experienced neuroradiologists (radiologist A and radiologist B, with 5 and 10 years of neuroimaging experience, respectively) who were blinded to the clinical data. This modification was accomplished using the following steps: (1) removal of nonbrain tissue, brainstem, and cerebellum and (2) modification of WM segmentation. Then, the WM volumes were imported into AK software (Quantitative Analysis Kit, version 1.2, GE Healthcare) for image preprocessing. First, all images were resampled to 1 × 1 × 1 mm^3^ resolution through linear interpolation to eliminate the effect of anisotropy on the features. A Gaussian filter was then applied to reduce noise, and the magnetic field inhomogeneity was corrected, which also assisted with reducing the effects of external interference factors. Finally, the intensity was standardized to limit the grayscale values of all images to a range of 0-32 and ensure that they would be compared without bias (Sun et al., 2018).

### Radiomic Feature Extraction

**Figure 2:**
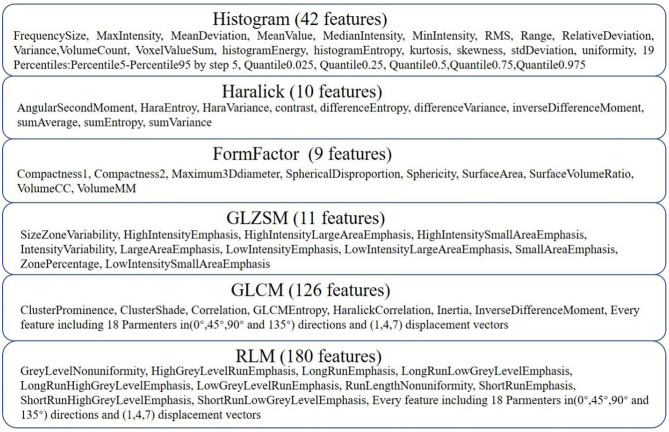
Details of the extracted features. Three hundred seventy-eight features were extracted from six categories.

AK software was used to extract 378 radiomic features based on the WM images, including histogram (42 features), Haralick (10 features), form factor (9 features), gray-level co-occurrence matrix (126 features, GLCM), run-length matrix (180 features, RLM) and gray-level size-zone Matrix (11 features, GLSZM) features. A detailed description of the features is provided in Figure 2. These features have been shown to characterize cancer heterogeneity and potentially reflect changes in the image structure (Mayerhoefer et al., 2020). In addition, we used the features that were most robust to manual correction by different radiologists (Shu et al., 2019) to ensure the stability and repeatability of the radiomics features. The Spearman rank correlation test was used to calculate the correlation coefficient (CC) of each feature between feature set A (from radiologist A) and feature set B (from radiologist B). Features with a CC> 0.8 were considered robust features (Wu et al., 2016). The quantitative value of robust features is the average of the two features.

### Establishment of an Overarching Radiomic Biomarker

Not every single feature is equally relevant to the diagnosis of PD. Furthermore, data reduction or feature selection is necessary to obtain meaningful results from pattern recognition analysis (Ashburner, 2009). In the present study, the minimum redundancy maximum relevance (mRMR) algorithm was used to extract robust features from the training set (Mukaka, 2012). The aim of the maximum relevance procedure was to select features with the maximal correlation with the actual PD diagnosis. At the same time, the minimum redundancy process ensured that the selected features had minimal redundancy among the other features, and we defined features with CCs greater than 0.1 and 0.8 as high-correlation and low-redundancy features, respectively. Then, the mRMR method was used to obtain an optimal feature set with a high correlation and low redundancy. Second, the least absolute shrinkage and selection operator (LASSO) algorithm was then applied to reduce the dimensionality of the optimal feature set. Finally, a gradient boosting decision tree (GBDT) algorithm was utilized to select radiomic features and build the composite radiomic biomarker. In order to quantify the radiomics biomarker discriminability, a score was calculated using the biomarker model from each patient in the training set. This result reflected the possibility of PD and was defined as the rad-score. The training set biomarker formula was employed to calculate the scores for the test set. Finally, the accuracy of the radiomic biomarker obtained from the training and test sets was evaluated by constructing a receiver operating characteristic (ROC) curve. To further verify the clinical efficacy of the radiomics biomarker, we conducted a stratified analysis of the rad-scores for patients with different nonmotor symptoms. Detailed information on dimensionality reduction is provided in the Supplementary Materials.

### Construction of the Integrative Nomogram

**Figure 3:**
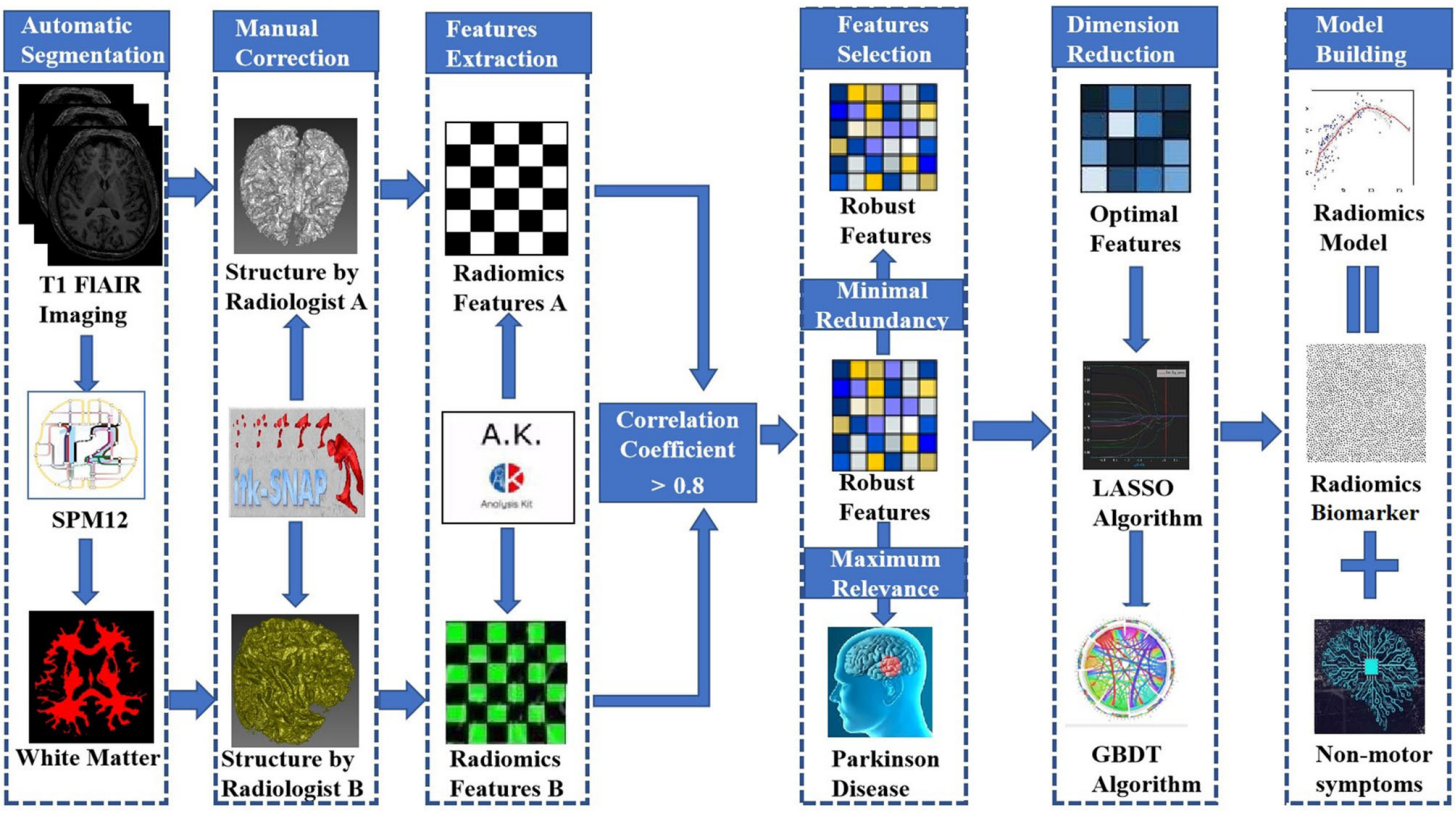
Workflow used to build the radiomic model.

Stepwise logistic regression analysis was performed to select independent predictors of PD for each potential predictive variable: demographic characteristics (i.e., family history of PD, age, and sex), nonmotor symptoms (i.e., impaired olfaction, depression, RBD, EDS, and CD) and radiomic biomarkers in the training set. In addition, machine learning, an important part of radiomics, improves the accuracy, performance, and predictive ability of the model (Chen et al., 2018; Watson et al., 2019). Accordingly, five machine learning classifiers were used to construct the predictive model and included support vector machine (SVM), Bayes, logistic regression, random forest, and decision tree classifiers. All models were examined using 5-fold cross-validation, in which 20% of the data were used to test the biomarker that was created with the other 80% of data. Different test and training set data were used for every 5-fold cross-validation, and the average classification accuracy was calculated based on 10 iterations of 5-fold cross-validation. The accuracy of the model using different machine learning classifiers was evaluated with an ROC curve and the DeLong test. Finally, the best machine learning method was applied to develop a predictive model for PD based on independent predictors, and an integrative nomogram was constructed. Figure 3 shows the workflow for creating the radiomics model. Detailed information about the machine learning techniques used in the present study is provided in the Supplementary Materials.

### Assessment of the Integrative Nomogram

Based on the nomogram, the risk score of PD was calculated for each patient. The accuracy of the nomogram obtained from the training and test sets was then evaluated with an ROC curve. A calibration curve was generated to evaluate the calibration performance, and the Hosmer-Lemeshow test was performed to analyze the goodness-of-fit of the nomogram. We attempted to distinguish the patients with PD in the SWEDD dataset to further evaluate the performance of the integrative nomogram. A dataset of age- and sex-matched patients with SWEDD and PD was collected from the PPMI, and the probability of PD was defined from the model score and was calculated for each patient using the integrative nomogram. Taking the threshold of the Youden index of the ROC curve as the classification point, we divided all cases into a low-risk and a high-risk group according to the model score. Based on the actual PD patients in different risk groups, the clinical effect of the nomogram was determined. Finally, the net benefit of the model was evaluated using decision curve analysis (DCA) (O'Brien, 2007).

### Statistical Analyses

Statistical analyses were performed with the Statistical Package for the Social Sciences (SPSS) version 22.0 (SPSS, Inc., Chicago, IL, USA), GraphPad Prism 6 (GraphPad Software, San Diego, CA, USA) and R software (version 3.3.1). Differences between categorical variables were examined using a chi-square test. Parametric data were assessed using an independent-sample *t*-test, whereas nonparametric data were assessed using the Mann-Whitney *U*-test. All analyses were controlled for age and sex. The nomogram was constructed, and calibration plots were generated using the “rms” package. The DCA was performed with the “dca.R.” package. Results with a two-tailed *P* < 0.05 were considered significant.

## Results

### Comparison of Patients' Clinical Data

Significant differences in Hoehn-Yahr staging were observed between patients in the three datasets (training set, test set and SWEDD set), but other clinical features were not significantly different, as shown in Table 1. However, a family history of PD, impaired olfaction and CD were significantly different between HCs and patients with PD in both the training and test sets, and RBD was significantly different between HCs and patients with PD in the test set. No other significant differences existed, as shown in Table 2.

**Table 1:** Descriptive statistics of the three datasets.

**Variables**		**Training set** **(*n* = 234)**	**Test set** **(*n* = 102)**	**SWEDD set** **(*n* = 58)**	***P*-value**
		***n*** **(%)**	***n*** **(%)**	***n*** **(%)**	
Sex	Male	146 (62.4)	64 (62.7)	37 (63.8)	0.981
	Female	88 (37.6)	38 (37.3)	21 (36.2)	
Age	Years	61.9 ± 9.7	61.6 ± 9.8	60 ± 9.7	0.399
Family history	No	196 (83.8)	88 (86.3)	43 (74.1)	0.129
of PD					
	Yes	38 (16.2)	14 (13.7)	15 (25.9)	
Impaired olfaction	No	92 (39.3)	33 (32.4)	25 (43.1)	0.336
	Yes	142 (60.7)	69 (67.6)	33 (56.9)	
Depression	No	211 (90.2)	85 (83.3)	45 (77.6)	0.023
	Yes	23 (9.8)	17 (16.7)	13 (22.4)	
RBD	No	171 (73.1)	70 (68.6)	36 (62.1)	0.232
	Yes	63 (26.9)	32 (31.4)	22 (37.9)	
EDS	No	191 (81.6)	84 (82.4)	45 (77.6)	0.738
	Yes	43 (18.4)	18 (17.6)	13 (22.4)	
CD	No	207 (88.5)	89 (87.3)	52 (89.7)	0.898
	Yes	27 (11.5)	13 (12.7)	6 (10.3)	
Hoehn-Yahr stage	Stage 0	106 (45.3)	47 (46.1)	3 (5.2)	<0.0001^*^
	Stage 1	54 (23.1)	21 (20.6)	30 (51.7)	
	Stage 2	74 (31.6)	34 (33.3)	25 (43.1)	

*SWEDD, scans without evidence of dopaminergic deficit; RBD, rapid eye movement sleep behavior disorder; EDS, excessive daytime sleepiness; CD, cognitive decline. P values, significance levels of differences in variables among the training set, test set and SWEDD set. The values in parentheses indicate the percentage of each variable in the training set or test set*.

* *P < 0.05*.

**Table 2:** Clinical characteristics of the training and test sets.

**Variable**	**Training set (*****n*** **=** **234)**				**Test set (*****n*** **=** **102)**		
	**HC (*****n*** **=** **117)**		**PD (*****n*** **=** **117)**		**HC (*n* = 51)**	**PD (*n* = 51)**	
		***n* (%)**	***n* (%)**	***P-*value**	***n* (%)**	***n* (%)**	***P-*value**
Sex	Male	75 (64.1)	71 (60.7)	0.589	31 (60.8)	33 (64.7)	0.682
	Female	42 (35.9)	46 (39.3)		20 (39.2)	18 (35.3)	
Age	Years	62.5 ± 9.9	61.4 ± 9.7	0.417	60.7 ± 9.1	62.1 ± 10	0.473
Family history of PD	No	105 (89.7)	91 (77.8)	0.013^*^	49 (96.1)	39 (76.5)	0.004^*^
	Yes	12 (10.3)	26 (22.2)		2 (3.9)	12 (23.5)	
Impaired olfaction	No	78 (66.7)	14 (12)	<0.0001^*^	27 (52.9)	6 (11.8)	<0.0001^*^
	Yes	39 (33.3)	103 (88)		24 (47.1)	45 (88.2)	
Depression	No	108 (92.3)	103 (88)	0.272	43 (84.3)	42 (82.4)	0.79
	Yes	9 (7.7)	14 (12)		8 (15.7)	9 (17.6)	
RBD	No	88 (75.2)	83 (70.9)	0.461	40 (78.4)	30 (58.8)	0.033^*^
	Yes	29 (24.8)	34 (29.1)		11 (21.6)	21 (41.2)	
EDS	No	99 (84.6)	92 (78.6)	0.237	41 (80.4)	43 (84.3)	0.603
	Yes	18 (15.4)	25 (21.4)		10 (19.6)	8 (15.7)	
CD	No	114 (97.4)	93 (79.5)	<0.0001^*^	51 (100)	38 (74.5)	<0.0001^*^
	Yes	3 (2.6)	24 (20.5)		0 (0)	13 (25.5)	

*RBD, rapid eye movement sleep behavior disorder; EDS, excessive daytime sleepiness; CD, cognitive decline. P-values, significance levels of differences in variables between the training and test sets. The values in parentheses indicate the percentage of each variable in the training set or test set*.

* *P < 0.05*.

### Development and Accuracy of the Radiomic Biomarker

**Figure 4:**
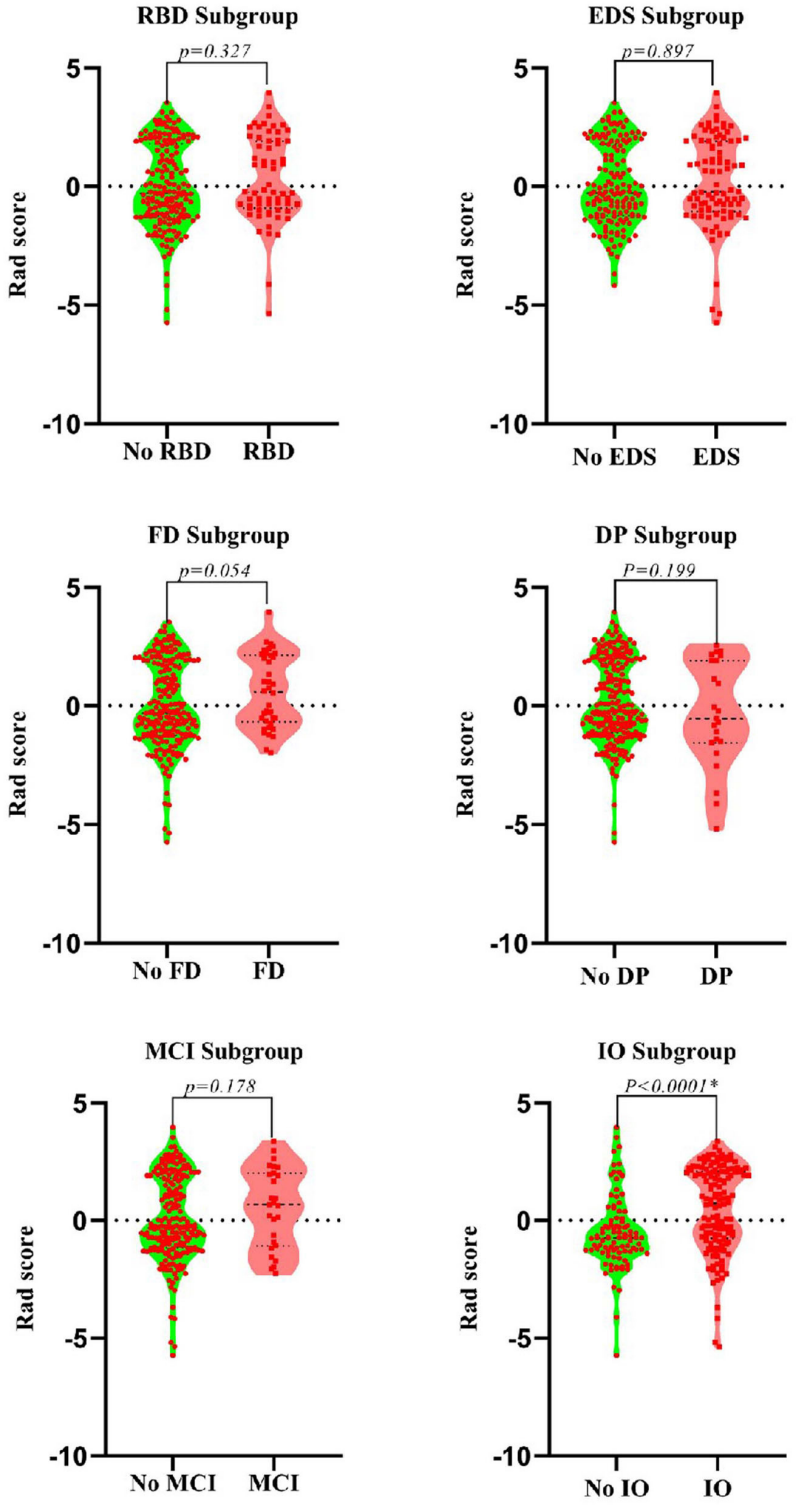
Violin plots of subgroups of patients stratified by non-motor symptoms. The blue line represents the median, and the red lines represent the first and third quartiles. RBD, rapid eye movement sleep behavior disorder; EDS, excessive daytime sleepiness; FD, family history of PD; DP, depression; CD, cognitive decline; IO, impaired olfaction.

After dimensionality reduction was applied to the 378 extracted features, four features were ultimately selected to construct the radiomics biomarker using logistic regression analysis. Detailed information on the dimensionality reduction process and features is provided in the Supplementary Materials. The rad-score was calculated from the formula for the radiomics biomarker, and it displayed favorable predictive efficacy in the training and test sets (the area under the curve (AUC) values were 0.838 and 0.826, respectively; the specificity was 83.8 and 84.3%, respectively; and the sensitivity was 71.8 and 74.5%, respectively). In addition, the nonmotor symptoms of patients with PD were compared using the rad-score. We observed a significant difference in rad-scores between patients with PD without olfactory disturbances and patients with PD and olfactory impairment, as illustrated in Figure 4.

### Development of an Integrative Nomogram

**Figure 5:**
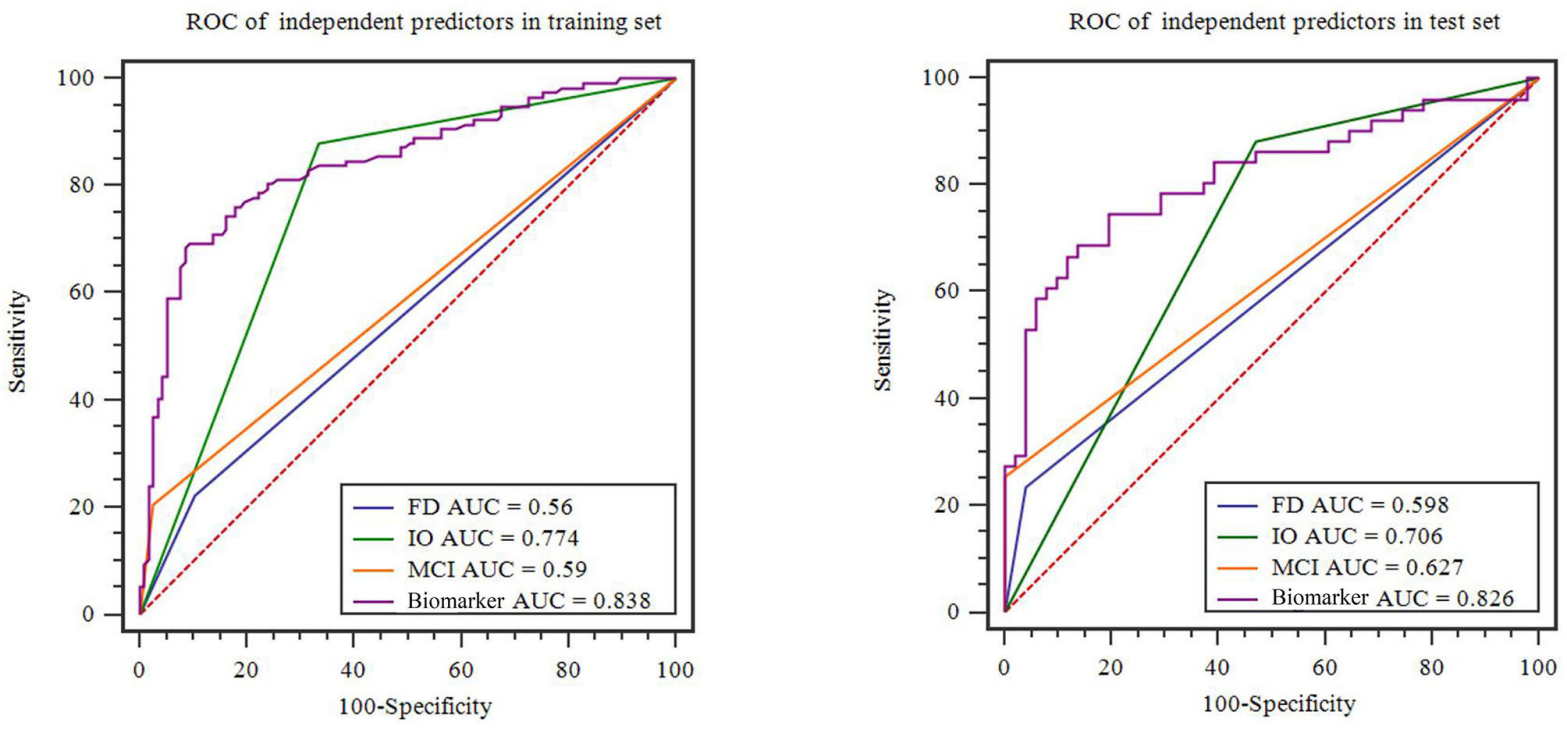
Diagnostic accuracy of the independent predictors in the training and test sets. FD, family history of PD; CD, cognitive decline; IO, impaired olfaction.

**Figure 6:**
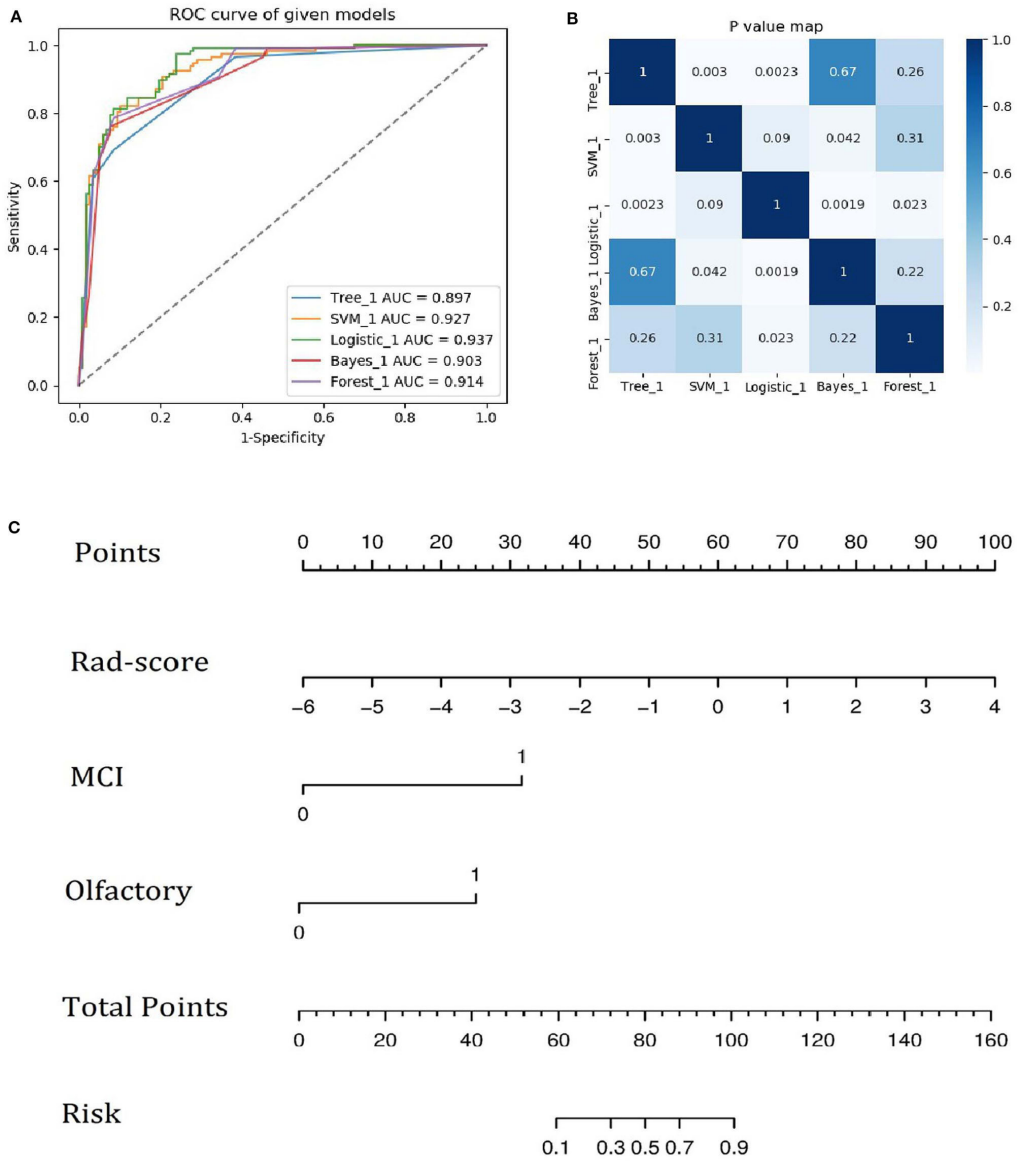
**(A)** ROC curves of the five machine learning methods. **(B)** Heatmap of *p*-values obtained using the model compared with each of the other machine learning methods based on the DeLong test. **(C)** Integrative nomogram used to detect PD. The nomogram was developed using the training set. In the nomogram, a vertical line is first generated according to the value of the rad-score to determine the corresponding score. Similarly, the scores for CD and olfaction are also determined. Then, the total score is calculated as the sum of the three scores described above. Finally, a vertical line is generated according to the value of the total score to determine the probability of PD.

A family history of PD, impaired olfaction, CD and radiomics biomarkers were independent predictors of PD according to the univariate logistic regression analysis. ROC curves showed that radiomics biomarkers had the highest diagnostic efficacy among these independent predictors (Figure 5). Then, impaired olfaction, CD and radiomics biomarkers were selected as the factors to construct the integrated model using a stepwise logistic regression analysis, as shown in Table 3. Based on three independent predictors, five machine learning methods were used to construct the model. The AUC values of the SVM, Bayes, logistic regression, random forest, and decision tree classifiers in the training set were 0.927, 0.903, 0.937, 0.914, and 0.897, respectively. The predictive performance of the different machine learning methods is presented in the Supplementary Material. The DeLong test showed a significant difference in the AUC value of the logistic regression model compared with the other machine learning methods. Accordingly, the logistic regression classifier was used to build the models and develop an integrative nomogram, as depicted in Figure 6.

**Table 3:** Stepwise logistic regression analysis of the nomogram for PD prediction.

**Variable**	**Univariate logistic regression analysis**		**Multivariate logistic regression analysis**	
	**OR (95% CI)**	***P*-value**	**OR (95% CI)**	***P*-value**
Sex (male vs. female)	1.237 (0.52–2.943)	0.631	NA	NA
Age (per 1-year increase)	0.97 (0.928–1.015)	0.19	NA	NA
Family history of PD (No vs. Yes)	2.5 (1.194–5.237)	0.015^*^	NA	NA
Impaired olfaction (No vs. Yes)	16.992 (6.51–44.352)	<0.0001^*^	16.251 (6.549–40.326)	<0.0001^*^
Depression (No vs. Yes)	1.557 (0.382–6.337)	0.537	NA	NA
RBD (No vs. Yes)	0.719 (0.277–1.863)	0.497	NA	NA
EDS (No vs. Yes)	1.297 (0.427–3.936)	0.646	NA	NA
CD (No vs. Yes)	23.783 (3.34–169.356)	0.002^*^	28.34 (4.441–180.874)	<0.0001^*^
Radiomic score (per 0.1 increase)	2.9 (2.108–3.99)	<0.0001^*^	2.934 (2.145–4.013)	<0.0001^*^

NA, not available, as the variable was not included in the multivariate logistic regression analysis; RBD, rapid eye movement sleep behavior disorder; EDS, excessive daytime sleepiness; CD, cognitive decline. The P value indicates whether the variable is an independent predictor of PD, and

* *represents P < 0.05*.

### Performance of the Nomogram

**Figure 7:**
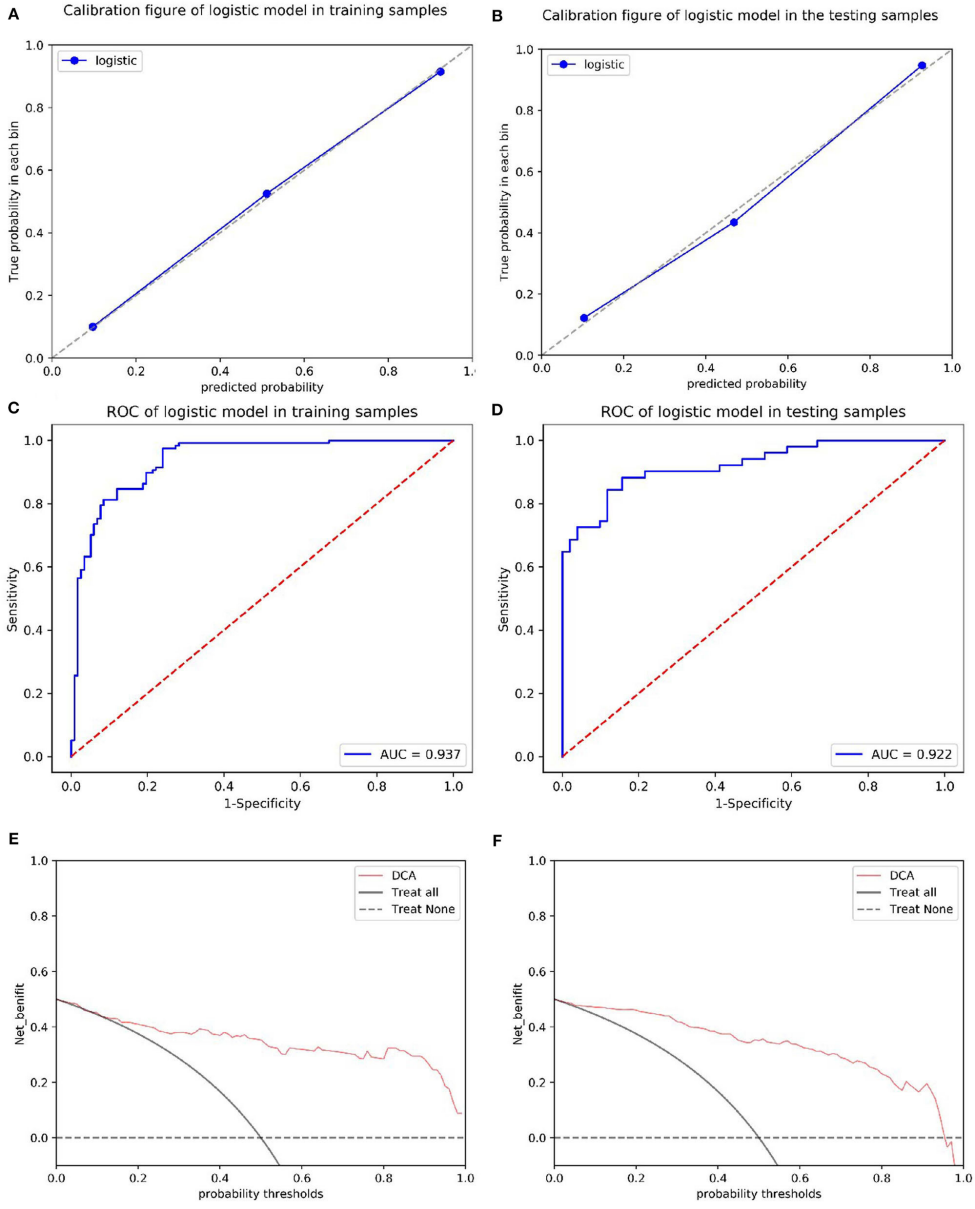
Calibration of the radiomics nomogram for PD in the training and test sets **(A,B)**. The dashed line represents the reference line where an ideal biomarker would lie, the dotted line represents the performance of the biomarker, and the solid line corrects for any bias in the biomarker. ROC curves of the radiomics nomogram for detecting the presence of PD in the training and test sets **(C,D)**. A DCA was performed to show the clinical effectiveness of the nomogram in predicting the presence of PD in patients included in the training and test sets **(E,F)**. The y-axis represents the net benefit. The pink line represents the radiomics nomogram. The solid black line represents the hypothesis that all patients had PD. The black dotted line represents the hypothesis that no patients had PD. The x-axis represents the threshold probability. The threshold probability is where the expected benefit of treatment is equal to the expected benefit of avoiding treatment. For example, if the possibility of PD in a patient is over the threshold probability, then a treatment strategy for PD should be adopted. The decision curves for the test set showed that if the threshold probability is between 0 and 0.88, then the use of the radiomic nomogram to predict PD provides a greater benefit than treating either all or none of the patients.

**Figure 8:**
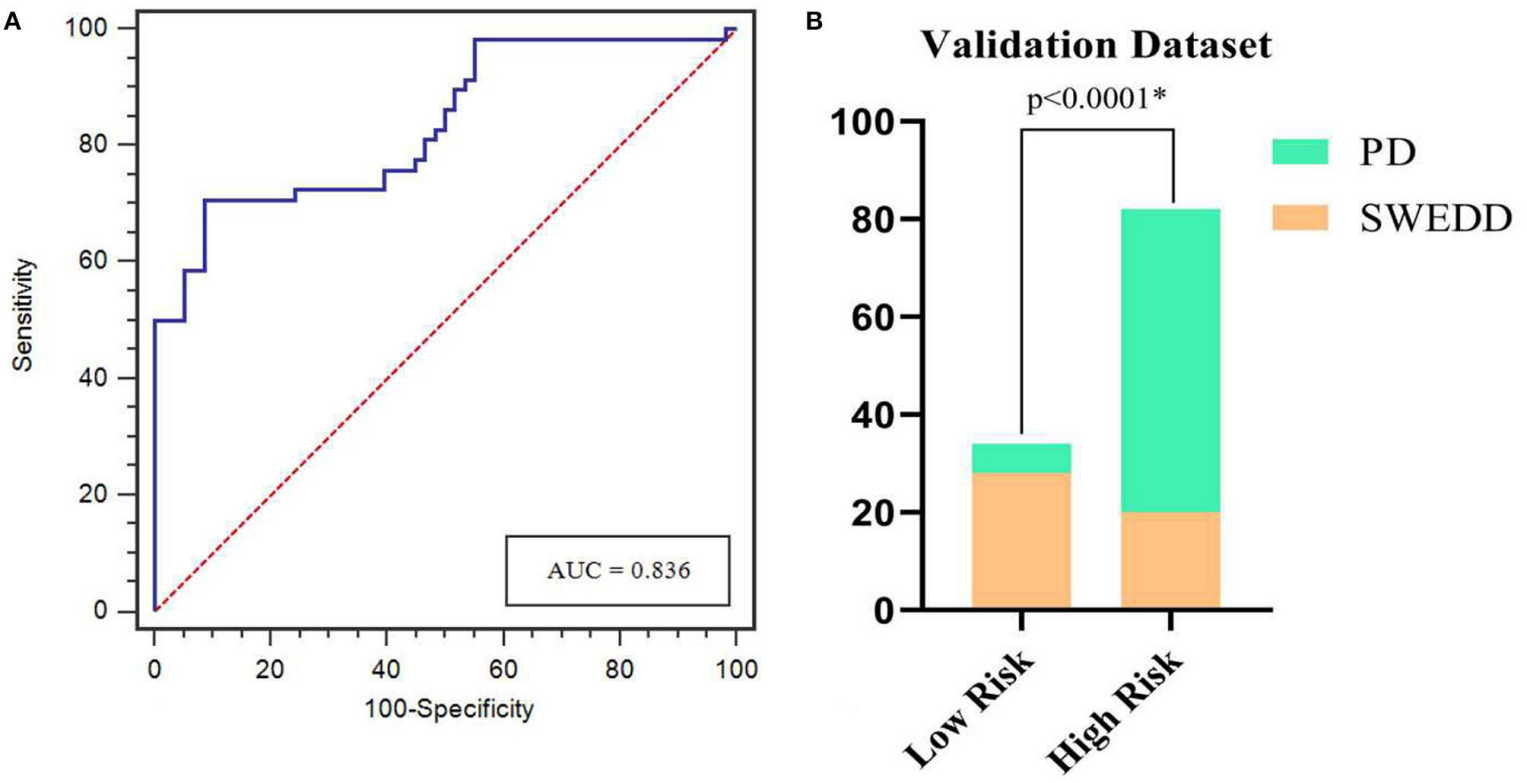
ROC curves for the nomogram in predicting PD in the validation dataset **(A)**. Bar plots show a significantly higher incidence of PD in the high-risk group than in the low-risk group **(B)**. *represents *p* < 0.05.

The calibration curves showed the consistency between the predicted PD probability and the actual PD probability for the nomogram in both the training and testing sets. The Hosmer-Lemeshow test did not reveal a significant difference between the performance of the nomogram in the training and testing sets (*P* > 0.05), indicating the lack of a deviation from the fit. The accuracy, specificity, and sensitivity of the nomogram for identifying PD were 0.937, 83.8, and 84.6%, respectively, for the training set and 0.922, 88.2, and 82.4%, respectively, for the test set. The DCA curves also showed good net benefits, which indicated the superior diagnostic accuracy of the nomogram, as indicated in Figure 7. The integrative nomogram showed good classification results in the datasets containing patients with PD and SWEDD from the PPMI. The AUC, sensitivity and specificity were 0.836, 70.69, and 91.38%, respectively. Finally, the dataset was divided into a high-risk group and a low-risk group according to the best diagnostic threshold of the nomogram (cutoff value: 0.2862), and the chi-square test revealed a significant difference in the number of patients with PD between the high-risk group and the low-risk group (χ^2^ = 40.474, ϕ = 0.5847, *P* < 0.001; Figure 8).

## Discussion

Our results show a difference in the value of radiomic biomarkers based on whole-brain white matter between patients with PD and HCs, suggesting that the microstructure of the WM in patients with PD is altered at the early disease stage (Rektor et al., 2018). Furthermore, the difference in the rad-score in the olfactory subgroup suggested that WM damage might be a risk factor for impaired olfaction. In addition, the integrative nomogram showed good performance for identifying patients with early-stage PD, particularly in the SWEDD dataset. We believe that the diagnostic model for PD will be expanded in the future, particularly given the convenience and speed of using the nomogram in the clinic.

Brain MRI is commonly used in clinical practice to evaluate the structural anatomy and pathology of the brain and is also used in the diagnostic workup of PD to exclude the presence of subcortical vascular pathology or other causes of secondary parkinsonism and to differentiate PD from atypical parkinsonism (Heim et al., 2017). However, conventional MRI does not increase the diagnostic value when the clinical diagnosis is uncertain, which is particularly true in the early stages of PD (Brooks, 2000; Meijer et al., 2012). Encouragingly, we were able to use T1-weighted images to identify patients with PD in the present study, which will further expand the application of conventional MRI sequences for the early diagnosis of PD. Similar studies using T2-weighted imaging (T2WI) from conventional MRI have constructed a radiomic model to distinguish patients with PD from HCs (Liu et al., 2020); however, the authors manually placed regions of interest at the caudate nucleus and putamen, which is a very subjective and time-consuming process. These structures are also very small, and the segmentation is not sufficiently accurate. Overall, imaging WM is very accessible and inexpensive in clinical practice compared to imaging of the caudate nucleus and putamen. In addition, we inferred that PD might tend to cause greater damage in WM than other neurological degenerative diseases, as evidenced by the excellent performance of the WM-based radiomics integrative nomogram for distinguishing patients with PD from patients with SWEDD. This increased performance may be due to WM changes that likely represent axonal degeneration and myelin damage, which often occur early in disease progression (Burke and O'Malley, 2013) and support our hypothesis. Interestingly, WM is not the main pathological substrate of PD, and the difference in radiomics features further confirms the existence of a compensatory mechanism in the brain tissue in response to early-stage PD (Mizuno et al., 2010), which will be studied in the future.

Adeli et al. combined MRI and SPECT and achieved a PD diagnostic accuracy of 97.5% (Adeli et al., 2017). Obviously, this figure exceeds the predictive accuracy reported in the current study, but the accuracy reported in the previous study mainly depended on the SPECT data, whereas the results of our study mainly depended on MRI data alone. In our results, the diagnostic efficiency of the radiomic biomarker based on MRI was 83.8%, much higher than the value for other nonmotor symptoms. Nonetheless, the diagnostic efficiency of radiomic biomarkers was lower in the present study than that the study by Wu et al. (2019), who showed that the diagnostic efficiency of radiomic biomarkers based on 18F-FDG PET images was 90.97%. However, because PET is not widely used in routine clinical practice, the method may lack practicality. Of course, we should not ignore the other factors that were used to build the model, including CD and impaired olfaction, which also contributed substantially to the model. In fact, impaired olfaction is one of the most common and typical nonmotor disorders associated with PD (Fullard et al., 2017), and most patients with PD develop an impairment of olfaction 4–6 years before they start to present motor impairment (Reichmann, 2017). This finding may also explain why olfactory impairment is the only non-motor symptom displaying statistically significant differences in the scores of the radiomics biomarker in patients with PD, and a deterioration in the sense of smell has been postulated to reflect extrastriatal neurodegeneration in patients with PD (Schrag et al., 2017). Accordingly, we speculate that olfactory damage may also reflect early changes in the WM microstructure, but further research is needed.

The advantages of the nomogram are also reflected in other aspects of this study. We analyzed 3D WM images, while most previous studies were based largely on a cross-sectional analysis of the SN (Takahashi et al., 2018; Cheng et al., 2019; Li et al., 2019). Nevertheless, a cross-sectional image of the SN may not completely reflect the early typical pathological changes associated with PD, and larger whole-brain changes will likely give a better representation of the global alterations associated with the disease. Four radiomic features were selected in the present study, including two features of RLM. In a previous study, RLM features, which reflect roughness and directionality, were also associated with the progression of white matter hyperintensity (WMH) (Li et al., 2017). Directionality refers to a specific route or angle of the nerve fasciculus. In normal WM, nerve fibers are properly oriented and regulated; nevertheless, when myelin is damaged, the neural structure may be disrupted (Yu et al., 2004), consistent with the white matter damage observed even in patients with early-stage PD in previous studies using advanced MRI technologies, such as DTI (Bergamino et al., 2020; Sanjari Moghaddam et al., 2020), showing that structural changes in the WM may underlie the clinical and pathologic heterogeneity of PD and cause relative cognitive impairment. Therefore, WM is a promising brain tissue to provide new insights that will be important for the early diagnosis of PD.

The correction curve and DCA showed the stability and clinical diagnostic benefits of the nomogram (AUC of 0.937), but the sensitivity of the nomogram may still be lower than that of cerebrospinal fluid, which was the first identified biomarker of PD (Olsson et al., 2016). As shown in a similar study, early diagnosis of PD based on a cerebrospinal fluid model constructed using machine learning achieved a sensitivity of 90% (Dos Santos et al., 2018). Regardless, the nomogram used in this study as a noninvasive evaluation tool may be more suitable for clinical application than invasive detection of cerebrospinal fluid. Furthermore, the accuracy of the nomogram decreased from 0.937 to 0.836 when applied to patients with SWEDD compared with HCs. We speculate that this decrease may be due to early microstructural changes in WM caused by other neurological diseases in patients included in the SWEDD dataset, and similar to patients with early-stage PD, these changes were accelerated. However, the difference between patients with PD and patients with SWEDD is likely attributable to the lack of dopaminergic neurons (Wyman-Chick et al., 2016); therefore, the model will undoubtedly further reflect the pathological mechanism of dopaminergic damage (Liu et al., 2018). Additionally, participants classified as having SWEDD who would later be diagnosed with PD might be distinguished from patients who would not develop PD. The nomogram was able to discriminate patients without evidence of dopaminergic deficits typical of PD from patients with other neurological disorders, which might be useful to clinicians, particularly when the nomogram is combined with imaging data.

Despite the overall positive results presented here, the current study still has some limitations. First, regarding the samples used for external verification, a larger sample size from multiple research centers is needed to verify and improve the results of the present study. Second, the patients with PD who were included in this study may have been in different neurological disease stages, and differences in WM features were still observed between patients with PD and HCs (the severity of PD did not appear to affect the results of this study). Final, we did not consider the possible effect of chronic dopaminergic medications on our results; the regimen of neuropsychiatric medications provided to patients during illnesses potentially affects brain structures (Zeng et al., 2015). Nevertheless, analysis of this cohort enabled us to establish a preliminary nomogram, facilitating the future consideration of long-term medication use in a larger and more diverse prospective study.

Although early diagnosis of PD is still based on clinical criteria, the advent of integrative nomograms will provide an imaging measure that can detect early-stage PD and may serve as a basis for future disease prediction studies in longitudinal cohorts.

## Data Availability Statement

Publicly available datasets were analyzed in this study. This data can be found at: http://www.ppmi-info.org.

## Ethics Statement

The study was approved by the institutional review board and the Zhejiang provincial ethics committee. Written informed consent for participation was not required for this study in accordance with the national legislation and the institutional requirements.

## Author Contributions

ZS, YX, and MZ contributed to the conception and design of the study. ZS, YX, SC, XW, and PP performed the data acquisition and analysis. ZS and YX wrote and revised the manuscript. All authors contributed to the article and approved the submitted version.

## Conflict of Interest

PP was employed by GE Healthcare. The remaining authors declare that the study was conducted in the absence of any commercial or financial relationships that could be construed as a potential conflict of interest.
