# Supplementary material for: An Integrative Nomogram for Identifying Early-Stage Parkinson's Disease Using Non-motor Symptoms and White Matter-Based Radiomics Biomarkers From Whole-Brain MRI
Source: Front Aging Neurosci. 2020 Dec 17;12:548616. doi: 10.3389/fnagi.2020.548616 (PMC7773758; doi:10.3389/fnagi.2020.548616)
Supplement: Supplementary file 1 [file Table_1.DOC]

**An integrative nomogram for identify early-stage Parkinson's disease using non-motor symptoms and MRI-based radiomic markers from whole brain white matter**

**I. Supplementary Methods**

1. **Research object matching**

We used the probability score matching in the spss22.0 software to analyze the gender and age of healthy controls (HC) and Parkinson’s disease (PD) patients. In the PPMI database, we first selected 189 HC and 347 PD patients, and then selected 168 HC and 168 PD patients after matching. In addition, we selected 168 PD patients and 60 scans without evidence of dopaminergic deficit (SWEDD) patients. 58 PD patients and 58 SWEDD patients were selected by matching. The result of the matching is shown in Figure S1.


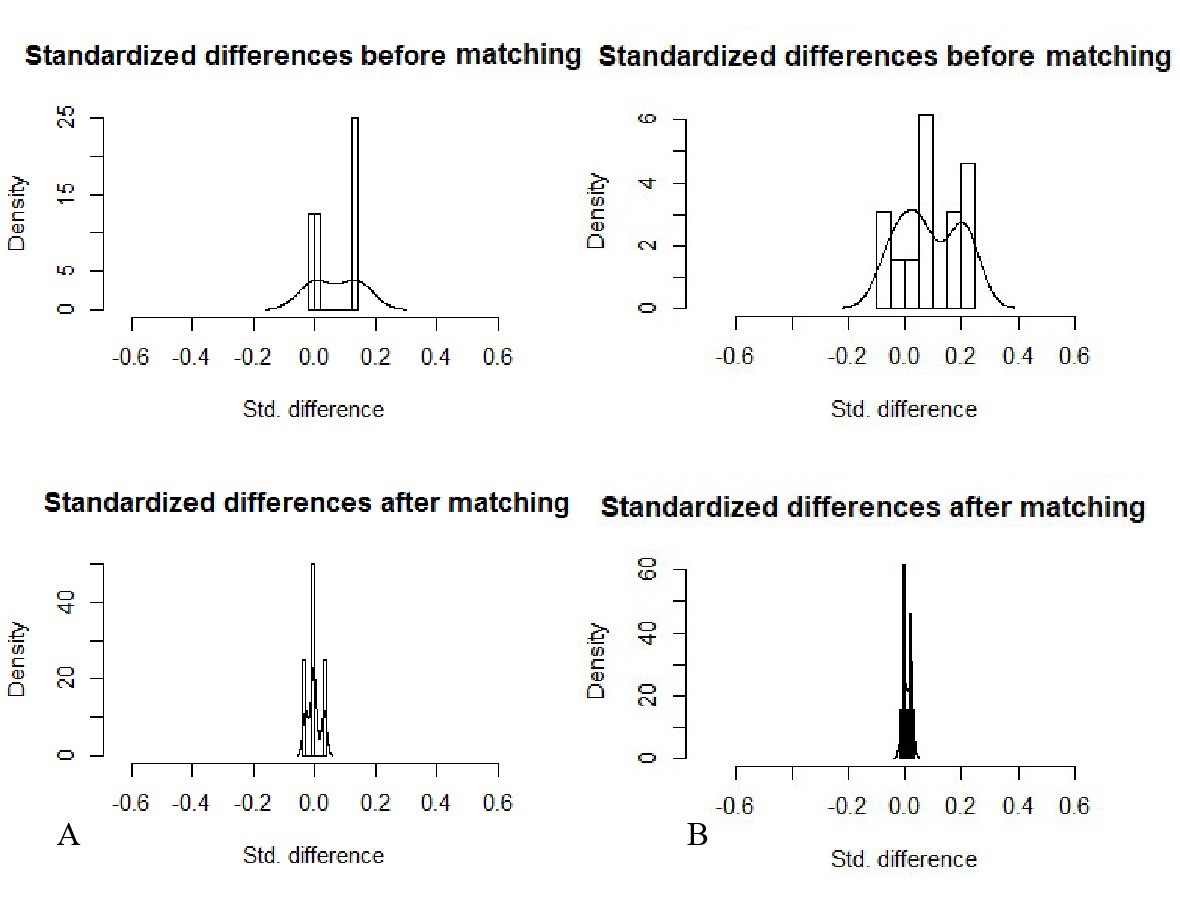


Figure S1. (A-B) Histogram of SD distribution. The difference in the datasets (PD vs HC and PD vs SWEDD) are concentrated around zero after matching gender and age. This indicates that the matching effect is excellent.

1. Disease duration for the PD patients

The mean interval time between the time of original data entry and time of PD diagnosis was 5.1 ± 6.3 months. The mean interval time between the time of original data entry and time of symptom was 21.6 ± 16.5 months. The mean interval time between the time of PD diagnosis and time of symptom was 16.5 ± 14.9 months. The result of the disease duration is shown in Figure S2. In addition, the sum of totle UPDRS score, include Parts I, II, III was 28.5 ± 15.8 from patients with PD. The result of details is shown in Figure S3.


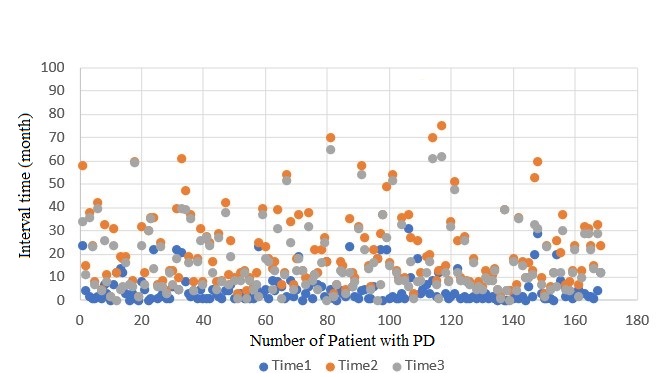


Figure S2. Scatter plot of disease duration in PD patients. The abscissa represents the PD patient serial number, and the ordinate represents the interval time. Time1: The mean interval time between the time of original data entry and time of PD diagnosis. Time2: The mean interval time between the time of original data entry and time of symptom. Time 3: The mean interval time between the time of PD diagnosis and time of symptom.


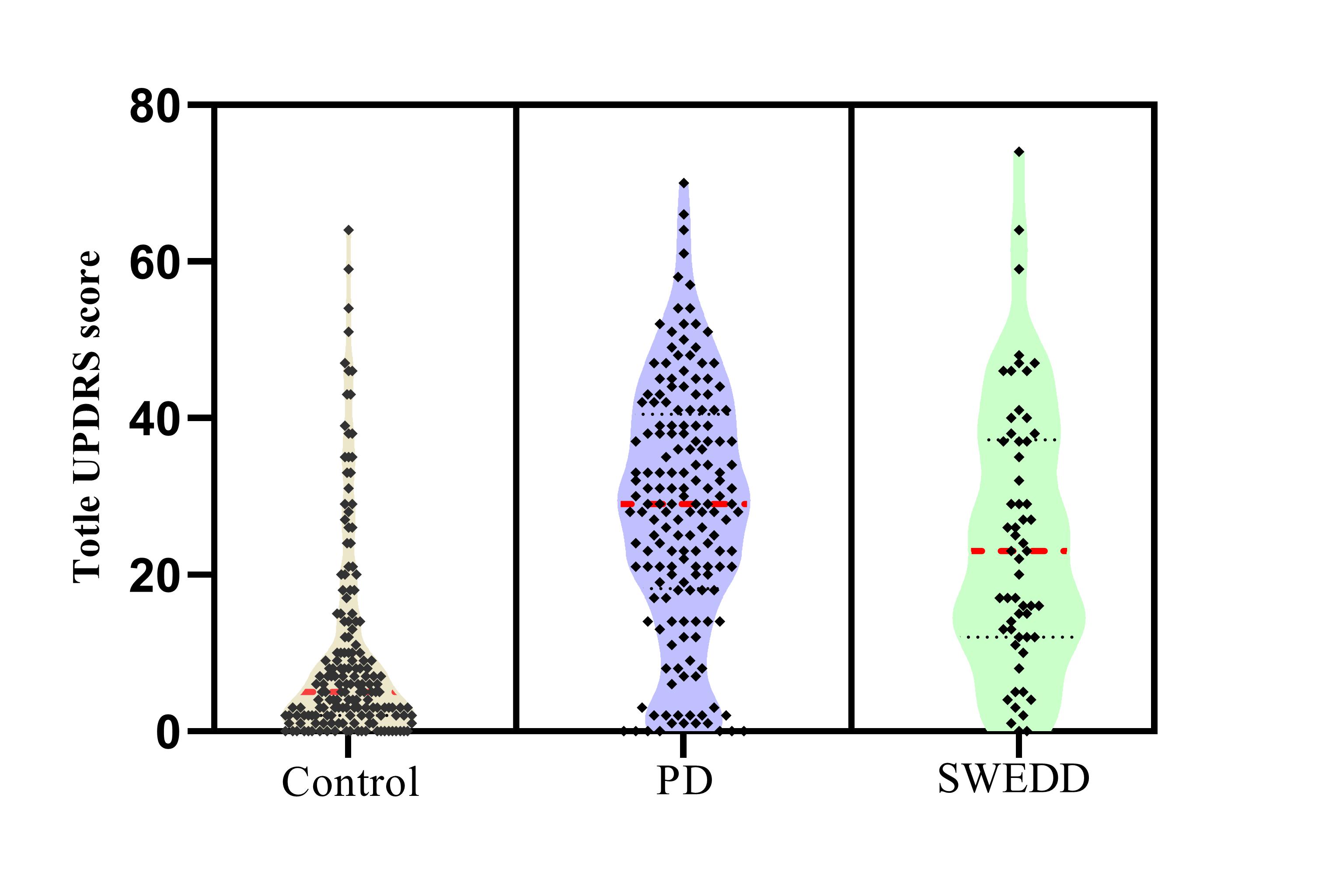


Figure S3.Total UPDRS violin diagram. The red dotted line represents the mean, and PD patients have the highest total UPDRS score in healthy people, PD and SWEDD groups.

1. **Neuropsychological assessment**

The Montreal Cognitive Assessment (MoCA) was performed to measure global cognitive function, and CD definedas MoCA an score < 26(adjusted for education) ^(1)^.

All smell testing used was done with the University of Pennsylvania Smell Identifi cation Test (UPSIT), which scores individuals from 0 to 40, and impaired olfaction definedas an UPSIT score < 36 ^(2)^.

Rapid eye movement sleep behavior disorder (RBD) is measured according to the screening questionnaire proposed in Stiasny-Kolster et al, and RBD definedas an score >4 ^(3)^.

Epworth Sleepiness Scale (ESS) is a standardized simple measure for sleep propensity, and EDS definedas an ESS score >9 ^(4)^.

The Geriatric Depression Scale(GDS) is based on a questionnaire of 30 items with binary outputs, and depression efinedas an GDS score > 4 ^(5)^.

**Ⅱ. Information of radiomics**

**1. Standardization of data**

Extracted texture features were standardized, which removed the unit limits of the data of each feature and converted it into a dimensionless pure value. This allowed the indexes of different units or orders to be compared and weighted. We used a z-score normalization to make the image intensities fit a standard normal distribution with $\mu\text{=0}$ and $\sigma\text{=1}$, where $\mu$ is the mean value of the images, and $\sigma$ is the standard deviation. The normalized values (also called z-scores) of the image intensities (*x*) were calculated as follows:

$$z=\frac{x-\mu}{\sigma}$$

1. **Details on Dimension reduction**

A total of 378 texture features were extracted from each patient, and 316 were retained by detection of robustness and reproducibility. The mRMR algorithm was used to select 139 features that had the greatest correlation with the outcome of PD, and then 20 features with the least redundancy were then selected in 139 features and build an optimal subset of complementary predictive features, Figure S4 shows the dimension reduction process of mRMR. Second, dimensionality reduction of the selected 20 features was performed using the least absolute shrinkage and selection operator (LASSO) method. LASSO is a powerful algorithm for regression analysis with high dimensional predictors. The LASSO algorithm shrinks some coefficients and reduces others to exactly 0 via an absolute constraint. Thus, LASSO is an outstanding method for feature selection as it retains good features using both a subset selection and ridge regression. In this study, LASSO selected 20 nonzero coefficients $\beta^{\wedge}$; Figure S5 shows the dimension reduction process of LASSO. Finally, the gradient boosting decision tree (GBDT) algorithm was used to reduce the dimension of the remaining features. GBDT is an algorithm that classifies or regresses data by the linear combination of basis functions and reduces the residual generated in the training process. In this study, four features were obtained from the GBDT procedure; details of these features are shown in Figure S6 and Table S1. In our study, the GBDT algorithm was combined with logistic regression to build the model as follows:

**alculation formula:**

Rad-score = 0.04579241+01.08305416×GLCMEntropy_angle135_offset1

-1.16995003×GreyLevelNonuniformity_angle45_offset7

-0.97908198×HaralickCorrelation_angle90_offset1

-0.14120719×HighGreyLevelRunEmphasis_AllDirection_offset7_SD

We used the GBDT-logistic model to construct a radiomics signature using the training set. Figure S6 shows the radiomics score of training and test sets using the GBDT-logistic model.


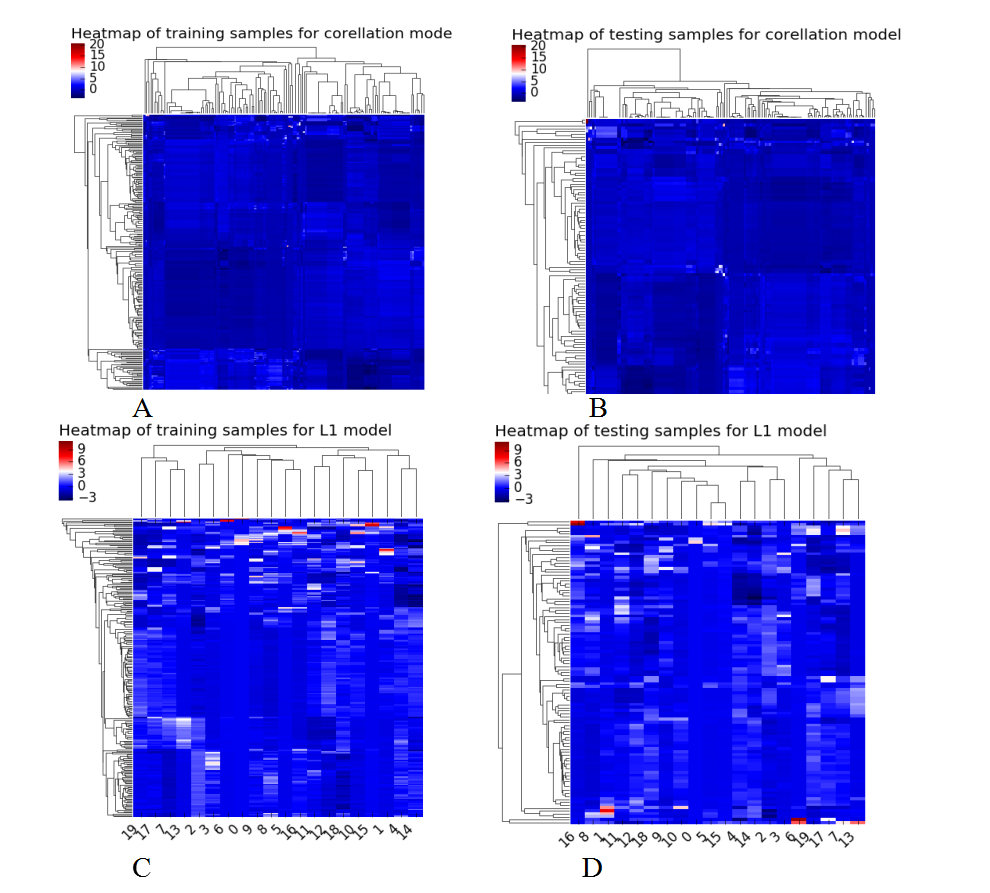


Figure S4. Process of mRMR for dimension reduction. Heatmap of the maximum correlation analysis between HC and PD in the training (A) and test (B) sets. Minimum redundancy feature heatmaps between HC and PD in the training (C) and test (D) sets.


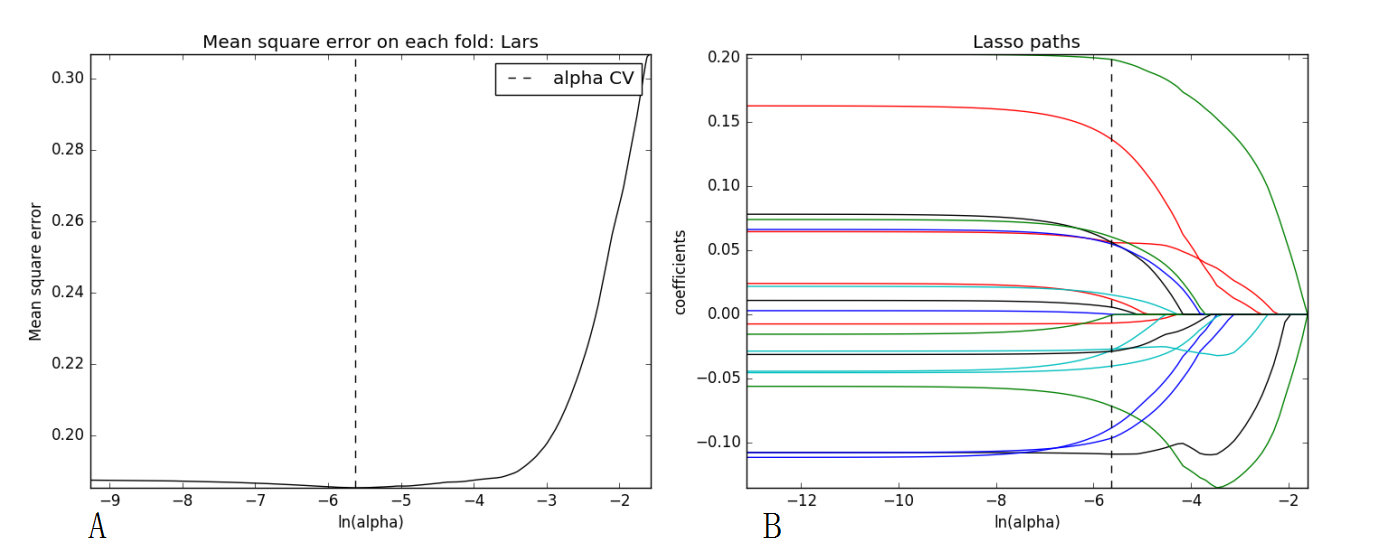


Figure S5. (A) Mean square error on each fold for the LASSO model. (B) LASSO path plot of the model in the training samples.


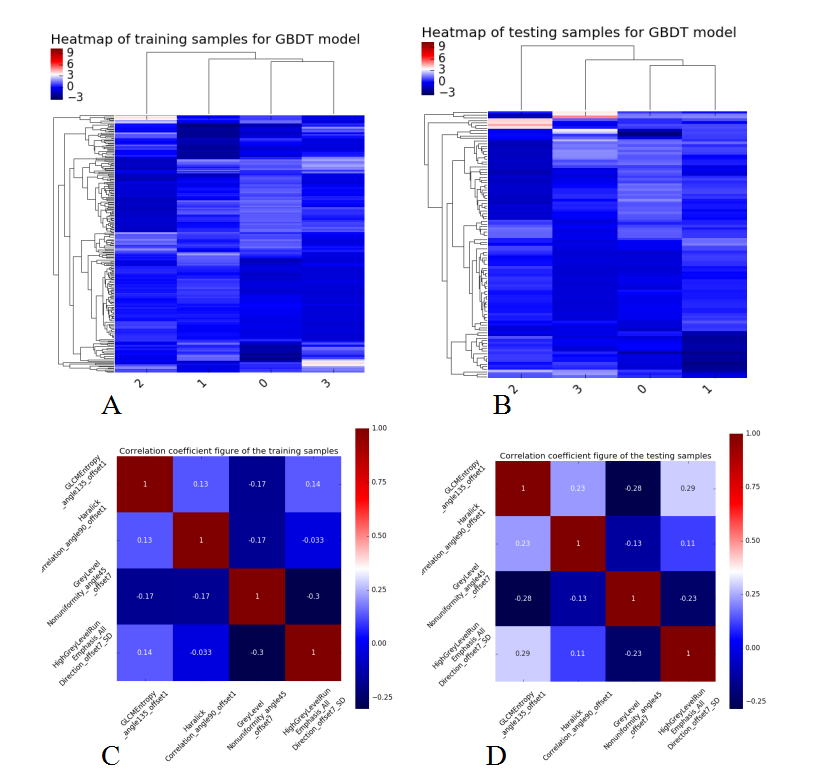


Figure S6. Process of GBDT for dimension reduction. Feature heatmap of the GDBT model in the training (A) and test (B) sets. The correlation coefficient of the training (C) and test (D) samples.


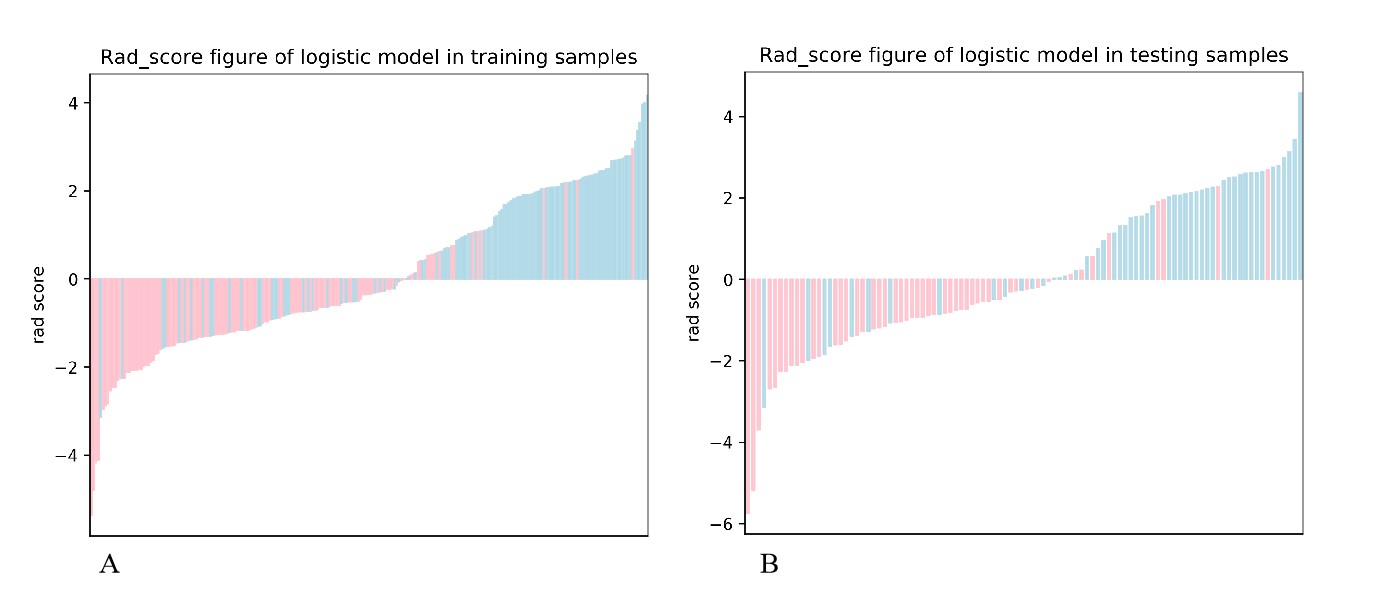


Figure S6. Score diagrams of the radiomics model in the training (A) and test (B) set. Red represents no PD and blue represents PD. A high score represents a high likelihood for PD. A score greater than 0 indicates PD, and a score less than 0 indicates no PD.

**Table S1. The classification and calculation formula of texture features**

| **Category** | **Feature** | **Formula** | **Describe** |
| --- | --- | --- | --- |
| RLM | GreyLevelNonuniformity_angle45_offset7 | 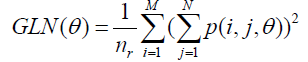 | The grey level run-length matrix (RLM) 𝐏𝐫(𝐢, 𝐣 | 𝛉 ) is defined as the numbers of runs with pixels of gray level i and run length j for a given direction θ. RLMs is generated for each sample image segment having directions (0°,45°,90° &135°), |
|  | HighGreyLevelRunEmphasis_AllDirection_offset7_SD | 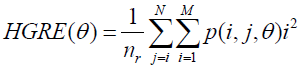 |  |
| GLM | GLCMEntropy_angle135_offset1 | 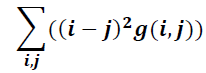 | Entropy shows the amount of information of the image that is needed for the image compression. Entropy measures the loss of information or message in a transmitted signal and also measures the image information |
| Haralick | HaralickCorrelation_angle90_offset1 | 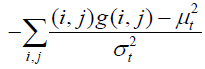 | Measures the degree of similarity of the gray level of the image in the row or column  direction. Represents the local grey level correlation, the greater its value, the greater the correlation |

**Table S2. Predictive performances of different machine learning methods**

| Group | Training set | | | | | Testing set | | | | |
| --- | --- | --- | --- | --- | --- | --- | --- | --- | --- | --- |
| Methods | SVM | Forest | Bayes | Logistic | Tree | SVM | Forest | Bayes | Logistic | Tree |
| Accuracy | 0.855 | 0.782 | 0.842 | 0.842 | 0.803 | 0.804 | 0.716 | 0.863 | 0.853 | 0.755 |
| F1 score | 0.85 | 0.806 | 0.828 | 0.843 | 0.779 | 0.787 | 0.76 | 0.848 | 0.848 | 0.706 |
| Recall | 0.821 | 0.906 | 0.761 | 0.846 | 0.692 | 0.725 | 0.902 | 0.765 | 0.824 | 0.588 |
| Precision | 0.881 | 0.726 | 0.908 | 0.839 | 0.89 | 0.86 | 0.657 | 0.951 | 0.875 | 0.882 |
| AUC | 0.927 | 0.914 | 0.903 | 0.937 | 0.897 | 0.901 | 0.88 | 0.885 | 0.922 | 0.831 |
| Sensitivity | 0.821 | 0.906 | 0.761 | 0.846 | 0.692 | 0.725 | 0.902 | 0.765 | 0.824 | 0.588 |
| Specificity | 0.889 | 0.658 | 0.923 | 0.838 | 0.915 | 0.882 | 0.529 | 0.961 | 0.882 | 0.922 |

**REFERENCES**

1. Goldman JG, Holden S, Ouyang B, et al. Diagnosing PD-MCI by MDS Task Force criteria: how many and which neuropsychological tests. Mov Disord. 2015, 30(3):402-6.
2. Rolheiser TM, Fulton HG, Good KP, et al. Diffusion tensor imaging and olfactory identification testing in early-stage Parkinson's disease. J Neurol. 2011, 258(7): 1254-60.
3. Stiasny-Kolster K, Mayer G, Schäfer S, et al. The REM sleep behavior disorder screening questionnaire--a new diagnostic instrument. Mov Disord. 2007, 22(16): 2386-93.
4. Johns MW. A new method for measuring daytime sleepiness: the Epworth sleepiness scale.Sleep. 1991, 14(6): 540-5.
5. Yesavage J, Brink T, Rose T, et al. Geriatric depression scale (GDS). Handbook of psychiatric measures.Washington DC: American Psychiatric Association, 544–6.
